# Mechanical Properties of Polypropylene Warp-Knitted Hernia Repair Mesh with Different Pull Densities

**DOI:** 10.3390/polym10121322

**Published:** 2018-11-29

**Authors:** Wanli Xu, Pibo Ma, Gaoming Jiang, Ailan Wan

**Affiliations:** 1 Engineering Research Center for Knitting Technology, Ministry of Education, College of Textile and Clothing, Jiangnan University, Wuxi 214122, China; wanlixu180509@163.com (W.X.); jgm@jiangnan.edu.cn (G.J.); ailanwan@163.com (A.W.); 2 State Key Laboratory of Bio-Fibers and Eco-Textiles, Qingdao University, Qingdao 266071, China

**Keywords:** hernia repair mesh, warp-knitted fabric, pull density, mechanical properties, stress

## Abstract

The medical polypropylene monofilament with a diameter of 0.10 mm was used as the material. Four different pull densities and two different warp run-ins were set up on the electronic traverse high-speed Tricot warp knitting machine, with the gauge of E28. The raw material was used to knit four variations of single bar plain knitted fabrics with 1 in-1 miss setting. Each variation required eight samples. The mechanical properties of the above 32 warp-knitted fabric samples are tested, including their tensile stress (in both vertical and horizontal directions), tearing stress (in both vertical and horizontal directions) and bursting stress. The results obtained shows that the relationship between the vertical, the horizontal stress, and the pull density are not monotonic. The tensile stress in the vertical direction firstly decreases and then increases with an increase of the pull density; however, the tensile stress in the horizontal direction firstly increases and then slightly decreases with an increase of the pull density; again the vertical tensile stress of all fabrics was always higher than the horizontal tensile stress. The bursting stress has a positive linear relation to the pull density. The vertical tearing stresses of four samples were greater than the horizontal tearing stress.

## 1. Introduction

Hernia has become a common disease in contemporary society. Failure to heal on time has serious consequences if left untreated. The surgical operation is a secure and effective way to treat hernia disease with a tensionless hernia repair mesh. This approach has many benefits such as repairing and strengthening the defective tissue, tension free, low recurrence rates, stability, short recovery period, simple to operate, and cost-effective.

The wide application and great economic benefit of hernia repair mesh produced by the warp knitting technology have aroused enormous attention into its research and development. Different stitches and materials have different functions in the hernia repair mesh. A gold wire was firstly used as a suture line and then woven with silver strands into a plain-woven mesh for the first hernia repair mesh by ancient Greeks [1]. Unfortunately, the gold mesh brought severe complications, including patient discomfort and material reabsorption. Later, nylon was woven into the hernia repair mesh with a plain-woven structure. Regrettably, nylon was not suitable for hernia repair due to the loss of strength with the hydrolytic digestion and needed explanation if infected [2]. Polypropylene was used as a hernia repair material due to its flexibility, strength, easy to cut, non-metallic, synthetic, non-absorbable, readily integrated by surrounding tissues, and resists inflection [3,4,5]. Some of the issues that are discussed include how selecting the hernia repair mesh, such as the physical and mechanical properties [6,7], and where the hernia repair mesh should be placed, such as the incisional hernia repair and laparoscopic hernia repair [8,9]. Hernia repair mesh will be directly in contact with tissue as an implantable medical device, so its performance directly affect the surgical effects and postoperative applications. The stiffness of the hernia repair mesh are mainly affected by the thickness of the mesh, the softer the hernia repair mesh is, the better fit the mesh will be, which contributes to tissue growth and recovery. However, it is not enough to resist the internal pressure of organization if the mesh is too thin. Usually, the thickness of hernia repair meshes is 0.6 mm. The pore size and porosity of the hernia repair mesh mainly affects the tissue growth, adhesion and anti-infective ability of the cells after it has been implanted in the human body. Surgical meshes used for hernia repair are sorted according to the pore size; large pore size allows the free entry of macrophages and contributes to nutrient absorption and waste excretion [10]. The areal density of the hernia repair mesh, that is the gram per square meter, relies on the amount of the material used and the effects on its flexibility and strength [11]. Hernia repair meshes with less than 66.67 g/m^2^ areal densities have better flexibility and are classified as light weight mesh. Hernia meshes with more than 66.67 g/m^2^ areal densities have larger tensile strength, larger surface area and are classified as heavy weight mesh; the greater weight of repair mesh would generate a greater foreign body reaction [12]. The difference of pore sizes and the oxidation via chronic inflammatory response also alter the mechanical properties [13,14]. Hernia repair mesh need to meet a certain mechanical performance index, such as tensile stress (transverse, longitudinal), tearing stress (transverse, longitudinal) and bursting stress, as the main role of the mesh is to resist the internal pressure of the human body and support the new tissue. Therefore, the biodegradability is not important. If the material has good biodegradability, it will degrade in humans and will not resist the pressure of the human body and cannot support the new tissue. However, hernia repair mesh also needs to meet a certain degree of biochemical performance, such as being non-toxic, stable property and good biocompatibility. Nanomaterials have aroused strong interest in the development of new biomedical applications [15,16]. The development of nanotechnologies contributes to numerous new products in various sectors. The stress-drive nonlocal elasticity model is crucial to study the structure of nanomaterial [17,18]. These relevant studies show the formulation of stress-driven nonlocal elasticity model and avoid basic difficulties of the classical strain-driven nonlocal model previously. Nanomaterial is becoming a developing trend in medical devices.

In this work, the design and production of hernia repair mesh are involved. The relationship between the mechanical properties of a hernia repair and its pull density, run-ins, and stitch structure are studied. The study is intended to lay a basis for its application in clinical medicine, and also help with the structural design or technical parameter design of hernia repair mesh in the future.

## 2. Experimental Section

### 2.1. Materials

**Figure 1:**
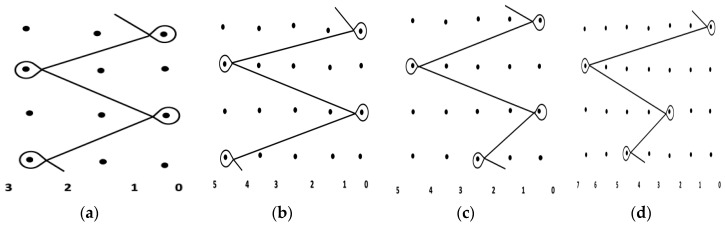
Stitch patterns used for (**a**) fabric 1#; (**b**) fabric 2#; (**c**) fabric 3# and (**d**) fabric 4#.

**Figure 2:**
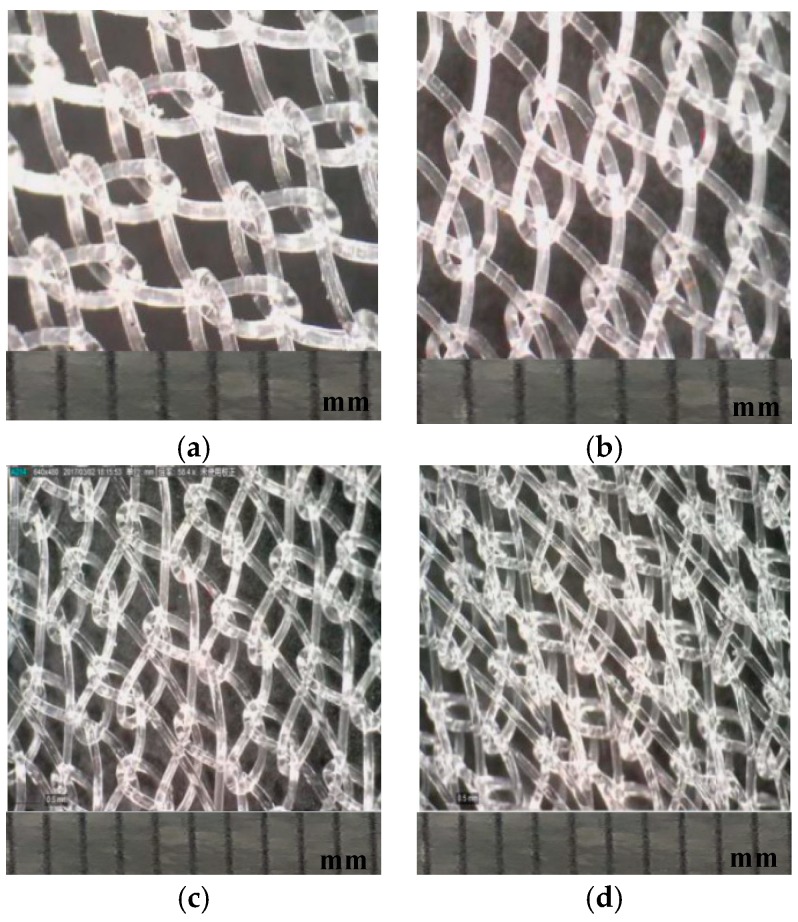
Actual knitted (**a**) fabric 1#; (**b**) fabric 2# (**c**); fabric 3# and (**d**) fabric 4# produced from stitch pattern (one scale value represents one millimeter).

The samples used for testing are mesh fabrics knitted by a Tricot warp knitting machine. The medical polypropylene monofilament with a diameter of 0.10 mm as raw material, which was firstly knitting on the SGE318 copy filament warp knitting machine. Then, four kinds of pull densities (8, 10, 12, 14 rows/cm) and the corresponding two kinds of warp run-ins (difference of 100 mm/rack) were set up on the electronic traverse high-speed Tricot warp knitting machine with the gauge of E28. The raw material polypropylene monofilament was used to knit four kinds of single bar plain knitted fabrics with 1 in-1 miss setting. The fabric 1# and fabric 2# is composed of three threads with a 3 × 1, five threads with a 5 × 1, respectively. The fabric 3# and Fabric 4# are a modified tricot stitch. Every variation required eight samples. The structure parameters of all fabrics are shown in Table 1. The stitch patterns of four fabrics are also shown in Figure 1, and the actual knitted patterns are shown in Figure 2. Finally, a heat setting process for 40 hernia repair meshes was carried out on R-3 baking type setting machine under 120 °C temperature for 40 s [14].

### 2.2. Mechanical Performance Test

All samples were tested at the temperature of 20 ± 2 °C with a humidity of 65 ± 2%.

#### 2.2.1. Tensile Property

**Figure 3:**
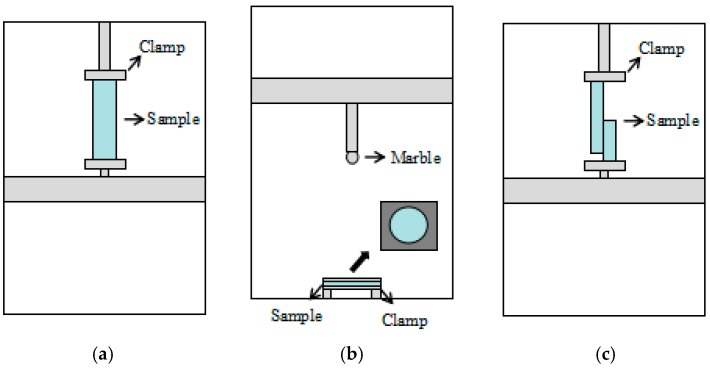
Schematic presentation of experimental tests (**a**) tensile test; (**b**) bursting test; (**c**) tearing test.

According to the international standard ISO 13934-1:1999 (Textiles-Tensile properties of fabrics-Part 1: Determination of maximum force and elongation at maximum force using the strip method), a multi-functional electronic fabric strength testing machine YG026A, as shown in Figure 3a, is used for the tensile testing with control programs and a computer for data collection. Samples were cut into the size of 6 × 30 cm (i.e., width and length, respectively) in both course-wise direction and wale-wise direction. Then pull the edge yarn along the wale direction making the size 5 × 30 cm (i.e., width and length, respectively). The tensile tests were conducted with the gauge of 100 mm, a tensile speed of 100 mm/min and a pretension of 2 N to acquire breaking strength. Each specimen was tested in the horizontal and vertical direction for five times, and the average figure was determined from the obtained results.

#### 2.2.2. Bursting Properties

According to the international standard ISO 3303-1990 (Rubber-or plastics-coated fabrics: Determination of bursting strength), all samples were tested on YG026D multi-functional electronic fabric strength testing machine as shown in Figure 3b. The samples were cut into a circular shape with a diameter of 60 mm. The diameter of the marble is 20 mm, with a descending speed of 100 mm/min. In addition, the gauge of the machine was set to 100 mm. Each sample was tested five times and the average figure was determined from the obtained results.

#### 2.2.3. Tearing Properties

With respect to the international standard ISO 13937-4-1995 (Textiles-Tear properties of fabrics-Part 4: Determination of tear force of tongue shaped test specimens), the tearing strength of all samples was conducted by using the tongue (single rip) method on a YG026A multi-functional electronic fabric strength testing machine as shown in Figure 3c. The specimens were cut into a rectangular shape with 100 cm along with the wale direction and 50 cm along with the course-wise direction. Then a 50 cm opening was cut with constitute 1/2 of the length of the specimen along the length direction. The tearing test was conducted with the gauge of 100 mm, a tensile speed of 100 mm/min and a pretension of 2 N. Each specimen was tested five times and the average figure was determined from the obtained results.

## 3. Results and Discussion

Tensile performance tests (in both vertical and horizontal direction), tearing performance tests (in both vertical and horizontal direction) and bursting performance test were carried out under the temperature of 20 ± 2 °C with a humidity of 65 ± 2%. The test results are shown in Table 2.

### 3.1. Effect of Pull Density on Tensile Properties

**Figure 4:**
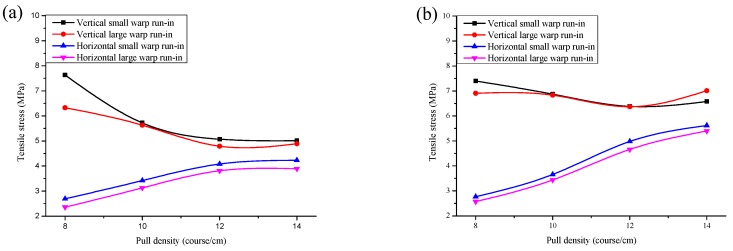

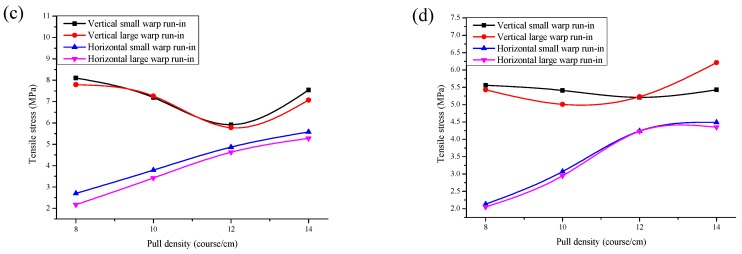
The relationship between the pull density and the vertical and horizontal tensile stress: (**a**) fabric 1#; (**b**) fabric 2#; (**c**) fabric 3# and (**d**) fabric 4#.

The relationship between the pull density and the tensile stress is shown in Figure 4. It can be seen from Figure 4 that the vertical stress of the fabrics is always higher than the horizontal stress, the reason is that the warp-knitted fabric is connected along the wale direction by loops but connected along the course direction by overlaps. The stress of connected loops is greater than overlaps. The relationship between the vertical, the horizontal stress, and the pull density are not monotonic. The tensile stress in the vertical direction firstly decreases and then increases with an increase of the pull density. The most tensile stress in the vertical direction is obtained in the pull density 8 course/cm. However, the tensile stress in the horizontal direction firstly increases and then slightly decreases with an increase of the pull density. With the increase of the pull density, the loop length of vertical and horizontal directions decrease, which make the course density and wale density more closer, so that the most tensile stress in the horizontal direction is obtained in a range of 12–14 (course/cm), and the gap of tensile stress between both the vertical and horizontal stress is smallest when the pull density is 12–14 (course/cm), which also indicates that the isotropy of the mesh is the best at that pull density. The tensile stress of fabric 3# in both the horizontal and vertical directions was higher than other samples, which could result from the relative larger overlaps and the symmetrically arranged overlaps. Thus the fabric 3# shows more excellent tensile properties.

### 3.2. Effect of Pull Density on Bursting Properties

**Figure 5:**
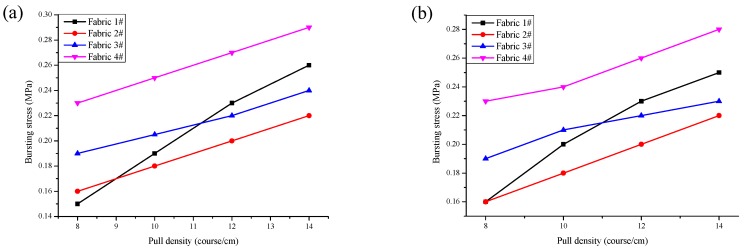
The relationship between the pull density and the bursting stress: (**a**) small warp run-in and (**b**) large warp run-in.

Figure 5 shows the relationship between the pull density and the bursting stress of all samples in different warp run-ins. The bursting stress increased obviously in linear relation with an increase in the pull density. The reason is that the loop length decrease, which makes the loop length decrease, then the mesh becomes smaller with an increase in the pull density during the knitting process, therefore the force of the yarn within a unit area increases. The bursting stress of fabric 1# had the most change with an influence from the pull density as shown in Figure 5. The reason is that the fabric 1# is designed by three threads with 3 × 1, the fabric with shortest average overlaps, which make the bursting stress of fabric 1# easily affected by pull density. The bursting stress of fabric 1# increased continuously with an increase in the pull density and reached a maximum at the pull density of 14 (course/cm). The bursting stress of fabric 4# was always higher than all the other fabrics, which is closely related to the longest overlap and complex distribution.

### 3.3. Effect of Pull Density on Tearing Properties

**Figure 6:**
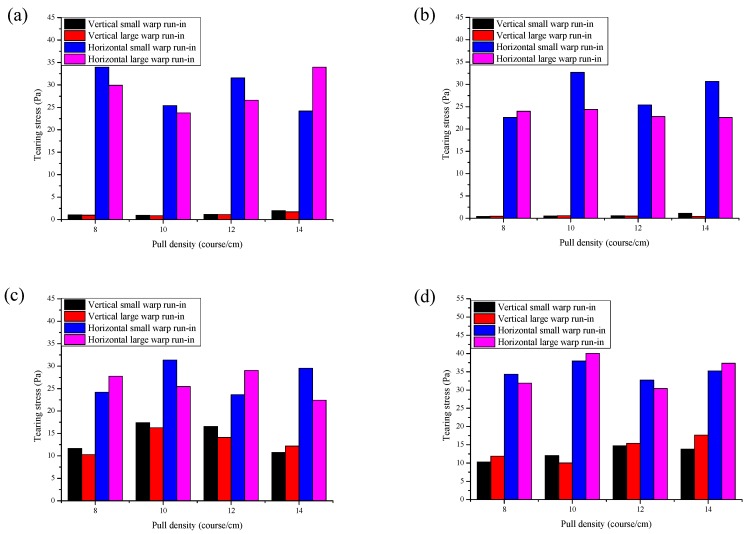
The relationship between the pull density and the vertical and horizontal tearing stress: (**a**) fabric 1#; (**b**) fabric 2#; (**c**) fabric 3#; and (**d**) fabric 4#.

Figure 6 shows the relationship between the pull density and the tearing stress under two kinds of warp run-ins. For a warp knitted fabric, it is interloped by loops in the vertical direction and connected by overlaps in the horizontal direction. As a result, the warp knitted fabric is easy to tear in the vertical direction. It can be seen from Figure 6 that the vertical tearing stress of all samples was always higher than the horizontal tearing stress, which is consistent with the theoretical speculation. The vertical tearing stress of fabric 1# and fabric 2# are below 2 Pa, while that of the other two stitches are greater than 10 Pa. The reason is that fabric 1# and fabric 2# are tricot stitch whose loops interlope each other on the same column and its overlaps are aligned, resulting in all loops laddering in turns after the first loop torn. So the vertical tearing stress of both fabric 1# and fabric 2# are relatively low. However, for fabric 3# and fabric 4#, their loops on the same column are not interloped by the same yarn because they are modified tricot stitches. Therefore the entire loops do not ladder when the first loop is torn. As a result, the vertical tearing stresses of all the stitches are relatively higher. In total, the vertical tearing stress is too low to apply in hernia repair mesh [10].

## 4. Conclusions

The mechanical properties of hernia repair mesh with various structure parameters are studied in this paper. It was found that the vertical stress of the fabrics is always higher than the horizontal stress. The relationship between the vertical, the horizontal stress, and the pull density are not monotonic. The tensile stress in the vertical direction firstly decreases and then increases with an increase of the pull density; however, the tensile stress in the horizontal direction firstly increases and then slightly decreases with an increase of the pull density. The bursting stress has a positive linear relationship with the pull density; there is no significant relationship between pull density and tearing stress. The isotropic property of the stitch mesh is the best when the pull density is 12–14 (course/cm). Under the same condition, the performance of fabric 3# is generally superior among all the four variations of samples. Due to the small vertical tearing stress, the ordinary tricot stitch with 1-in-1 miss setting by single bar is not suitable for the development and application of hernia repair mesh, but this can be improved using modified tricot stitch. Therefore, it is helpful in order to design hernia repair mesh with better mechanical properties through changing the structure and material parameters.

## Figures and Tables

**Table 1:** Structure parameters.

No.	Setting	Drawing	Pull Density (course/cm)	Warp Run-in (mm/rack)	Porosity (%)	Gram Weight (g/cm^2^)	Thickness (mm)
1-11	2-3/1-0//	1 miss-1 in	8	2500	37.0	95.1	0.56
1-12			8	2600	36.6	92.3	0.62
1-21			10	2200	36. 5	98.1	0.56
1-22			10	2300	36.1	94.3	0.58
1-31			12	2100	35.5	10.3	0.55
1-32			12	2200	34.8	10.2	0.57
1-41			14	2000	33.4	92.1	0.58
1-42			14	2100	33.0	90.2	0.59
2-11	4-5/1-0//	1 miss-1 in	8	3200	51.4	48.1	0.54
2-12			8	3300	50.3	48.0	0.55
2-21			10	3000	48.1	78.1	0.54
2-22			10	3100	47.4	77.8	0.54
2-31			12	2900	46.4	79.9	0.55
2-32			12	3000	45.3	79.1	0.56
2-41			14	2800	43.2	81.2	0.56
2-42			14	2900	42.9	80.6	0.57
3-11	2-3/1-0/	1 miss-1 in	8	2900	48.0	86.8	0.57
3-12			8	3000	46.1	86.1	0.56
3-21			10	2700	45.1	89.0	0.54
3-22			10	2800	44.	88.2	0.53
3-31	4-5/1-0//		12	2500	39.0	89.7	0.52
4-32			12	2600	36.6	89.1	0.52
3-41			14	2400	35.9	91.2	0.50
3-42			14	2500	34.8	90.8	0.51
4-11	4-5/3-2/	1 miss-1 in	8	3200	51.2	10.0	0.67
4-12			8	3300	50.2	10.0	0.66
4-21			10	2900	47.3	10.1	0.63
4-22			10	3000	45.0	10.1	0.64
4-31	6-7/1-0//		12	2800	40.7	10.8	0.60
4-32			12	2900	39.5	10.2	0.60
4-41			14	2700	38.1	11.0	0.59
4-42			14	2800	37.3	10.8	0.60

**Table 2:** The test results of all fabrics.

No.	Pull Density (course/cm)	Warp Run-in (mm/rack)	Vertical Tensile Stress (MPa)	Horizontal Tensile Stress (MPa)	Vertical Tearing Stress (Pa)	Horizontal Tearing Stress (Pa)	Bursting Stress (MPa)
1-11	8	2500	7.63	2.69	1.03	33.97	0.15
1-12	8	2600	6.33	2.36	0.99	29.92	0.16
1-21	10	2200	5.72	3.42	0.93	25.40	0.19
1-22	10	2300	5.63	3.13	0.85	23.78	0.20
1-31	12	2100	5.07	4.08	1.15	31.57	0.23
1-32	12	2200	4.79	3.81	1.11	26.57	0.23
1-41	14	2000	5.01	4.23	1.99	24.18	0.26
1-42	14	2100	4.89	3.89	1.72	33.97	0.25
2-11	8	3200	7.40	2.77	0.43	22.60	0.16
2-12	8	3300	6.91	2.57	0.46	23.97	0.16
2-21	10	3000	6.87	3.66	0.51	32.69	0.18
2-22	10	3100	6.83	3.44	0.57	24.39	0.18
2-31	12	2900	6.38	4.98	0.55	25.40	0.20
2-32	12	3000	6.37	4.66	0.50	22.79	0.20
2-41	14	2800	6.58	5.62	1.12	30.68	0.22
2-42	14	2900	7.01	5.40	0.43	22.60	0.22
3-11	8	2900	8.10	2.70	11.68	24.18	0.19
3-12	8	3000	7.79	2.17	10.29	27.75	0.19
3-21	10	2700	7.19	3.79	17.41	31.36	0.205
3-22	10	2800	7.26	3.42	16.28	25.45	0.21
3-31	12	2500	5.92	4.87	16.55	23.64	0.22
3-32	12	2600	5.78	4.63	14.13	29.02	0.22
3-41	14	2400	7.54	5.58	10.76	29.54	0.24
3-42	14	2500	7.07	5.28	12.18	22.41	0.23
4-11	8	3200	5.56	2.13	10.28	34.34	0.23
4-12	8	3300	5.43	2.05	11.90	31.91	0.23
4-21	10	2900	5.41	3.07	12.04	37.96	0.25
4-22	10	3000	5.01	2.95	10.04	40.06	0.24
4-31	12	2800	5.21	4.24	14.73	32.70	0.27
4-32	12	2900	5.23	4.23	15.40	30.42	0.26
4-41	14	2700	5.43	4.49	13.85	35.25	0.29
4-42	14	2800	6.21	4.35	17.67	37.34	0.28
